# Stimulus-Responsive Polymers Based on Polypeptoid Skeletons

**DOI:** 10.3390/polym13132089

**Published:** 2021-06-24

**Authors:** Rui Fang, Junwei Pi, Tiantian Wei, Amjad Ali, Li Guo

**Affiliations:** Research School of Polymeric Materials, School of Materials Science & Engineering, Jiangsu University, Zhenjiang 212013, China; 2211905015@stmail.ujs.edu.cn (R.F.); 2211905038@stmail.ujs.edu.cn (J.P.); 2211905055@stmail.ujs.edu.cn (T.W.)

**Keywords:** stimulus-responsive, polypeptoid, biomaterials

## Abstract

Polypeptoids have attracted a lot of atteSDntion because of their unique structural characteristics and special properties. Polypeptoids have the same main chain structures to polypeptides, making them have low cytotoxicity and excellent biocompatibility. Polypeptoids can also respond to external environmental changes by modifying the configurations of the side chains. The external stimuli can be heat, pH, ions, ultraviolet/visible light and active oxygen or their combinations. This review paper discussed the recent research progress in the field of stimulus-responsive polypeptoids, including the design of new stimulus-responsive polypeptoid structures, controlled actuation factors in response to external stimuli and the application of responsive polypeptoid biomaterials in various biomedical and biological nanotechnology, such as drug delivery, tissue engineering and biosensing.

## 1. Introduction

Responsive polymer materials can respond to changes in the external environment [1], such as heat [2], pH [3], ions, electric field [4], magnetic field [5] and ultraviolet/visible light [6], resulting in the change of their physical and chemical properties. Stimulus-responsive polymers are usually able to produce conformational changes induced by the changing environment to generate reversible microphase separation or self-assembly. These unique properties make scientists pay great attention to stimulus-responsive polymers, especially biopolymers that can be applied in drug delivery [7,8,9], tissue engineering [10,11] and sensing [12,13] systems. The research focuses on designing materials with good biocompatibility, low cytotoxicity and response to specific biological stimuli. 

Polysarcosine is the polypeptoid with great water solubility, making it an outstandingly interesting candidate for the synthesis of amphiphilic block copolymers. Polysarcosine is poly(N-methyl glycine) (PNMG). It was reported first by Wesseley and coworkers in the 1920s and found good biological properties [14]. Interestingly, in early publications, peptides and peptoids were not separated [15]. In 1992, Paul A. Bartlett defined oligomers of N-substituted glycines as peptoids [16]. Later, Ronald N. Zuckermann introduced the term polypeptoids for 30–36 residue long peptoids, thus establishing the commonly accepted terminology. In recent years, with the improvement of synthesis methods [17], polypeptoids have attracted more and more scientists for their great biocompatibility and other excellent properties. Many researchers have focused on applying polypeptoid structures in the biostimulation response. 

**Figure 1:**
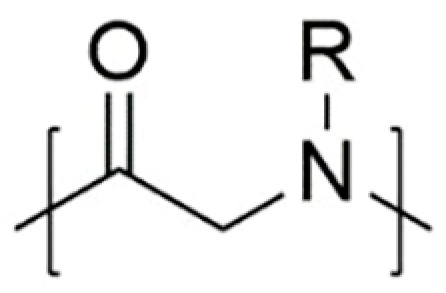
Schematic diagram of the chemical structure of polypeptoid.

Polypeptoids have low cytotoxicity and good biocompatibility because of their structural similarity to polypeptides (Figure 1) [18,19,20,21,22,23]. Different from polypeptides, however, polypeptoids show thermal transformation similar to synthetic thermoplastics, making them suitable for various heat treatment methods [24]. Polypeptoids combine the properties of natural macromolecules and synthetic polymers, and become great candidates as stimulus-responsive biopolymers. Polypeptoids can achieve their responsiveness to environmental stimuli by linking different functional groups to backbone nitrogen atoms as side chains [25,26]. Nowadays the research on stimulus-responsive polypeptoids mainly focuses on temperature, pH, light and reactive oxygen sensitivity. This review will discuss the recent advances on stimulus-responsive polypeptoids, from synthesis, properties to applications.

## 2. Polymeric Responsive Systems

With specific external stimulation, the macromolecular morphology and conformation of the polymers change accordingly, leading to the change of the physical and chemical properties of the polymers. 

In a particular solvent, usually in water, a temperature-responsive polymer is soluble in a specific temperature range, and too high or too low temperature will lead to insolubility. Therefore, the material has a lower critical solution temperature (LCST) or an upper critical solution temperature (UCST). In both cases, the phase behavior is determined by the interactions between polymer-polymer and polymer-solvent. Generally speaking, temperature-responsive polymers have components whose solubility varies with temperature change. The polymer can form micelles consist of a hydrophilic shell and a responsive hydrophobic core with adjustable solubility. External stimulation can cause the polymer micelles to undergo a reversible phase transition. Polymers with tunable LCST have attracted more and more attention in biological applications, such as biological separation, intelligent surface, drug release, targeted drug delivery, etc. [27,28]. These polymers are soluble below LCST, but phase separation occurs when heated above LCST. For example, the temperature-responsive triblock copolypeptoid, poly (N-allyl glycine) -b-Poly (N-methyl glycine)-b-Poly (N-decyl glycine), exhibits a sharp phase transition to temperature in water. The polymer is soluble in water below LCST through the hydrogen bonding with water molecules. When the temperature increases, the block with allyl chains undergoes cloud point temperature (T_cp_) transition due to dehydration to form a hydrophobic domain [29]. Even though most temperature-responsive polymers have LCST, some polymers exhibit UCST characteristics [30,31,32,33,34], such as polypeptoid modified by thioglycolic acid. The polymer is soluble in water at the higher temperature. At low temperature, the content of protonated moiety increases and the polymer solubility reduces. This is because at high temperature, the phase transition is driven by thermally controlled reversible hydrogen bonds, making the polymer water-soluble [35]. The phase transition temperature can be controlled by changing the molecular weight, end group structure and copolymer composition [36,37,38]. The polymer concentration and additives (such as inorganic salts) also affect the phase transition temperature [39,40,41]. Due to the interactions between polymer and solvent, LCST decreases with the increase of molecular weight. It has been reported that the introduction of short hydrophilic outer blocks with DP 1 to DP 3 increased the LCST. For example, Winnik et al. reduced the T_cp_ of oxazoline from 48.1 °C to 32.5 °C by coupling hydrophobic n-octadecyl groups to polymer chain ends [42].

PH is another commonly used external stimulus [43]. The unique property of pH-responsive polymers is that the adjustment of pH value can easily cause the change of interactions between ions and hydrogen bonds, resulting in reversible microphase separation or self-organization [3]. The groups containing weak acids and bases, such as carboxylic acid, phosphoric acid and amine, will change in ionized state according to different pH values. Polymer conformation and solubility can change with pH when the ionizable groups are linked to polymer structures. For example, carboxylic acid modified polypeptoids can easily dissolve in alkaline solution but will become insoluble when pH is reduced. It is because that more and more COO^−^ groups become protonated with the decrease of pH value, making the solubility of the polymer reduce and the solution become turbid [35]. PH-responsive polymers can be used for pH triggered drug release and biosensor [44].

Till now, temperature and pH response are the two most widely studied response modes of stimulus-responsive polymers. However, due to the unique advantage that light can be controlled in time and space, the photo response system is also desirable [45,46,47]. In addition, the photochemical reaction is considered a green synthesis pathway because photons do not leave residues [48]. Some specific structures can exhibit a light viscosity effect under ultraviolet irradiation, resulting in conformational contraction. Studies have been made to prepare photo-responsive polymers by using this phenomenon. Photo-responsive polymers usually need to introduce corresponding functional side chains, such as diarylethene, o-nitrobenzyl and azobenzene [49,50,51,52,53].

Recently, some researchers have developed dual response polymers by combining temperature response with pH response [54]. The temperature response can also be combined with the photo response [55,56]. These kinds of materials have a bright application prospect in the biocatalysis [57], drug carrier [58], coatings for protein adsorption [59], signal control of sensors and micro engines [60].

## 3. Stimuli-Responsive Polypeptoid

**Figure 2:**
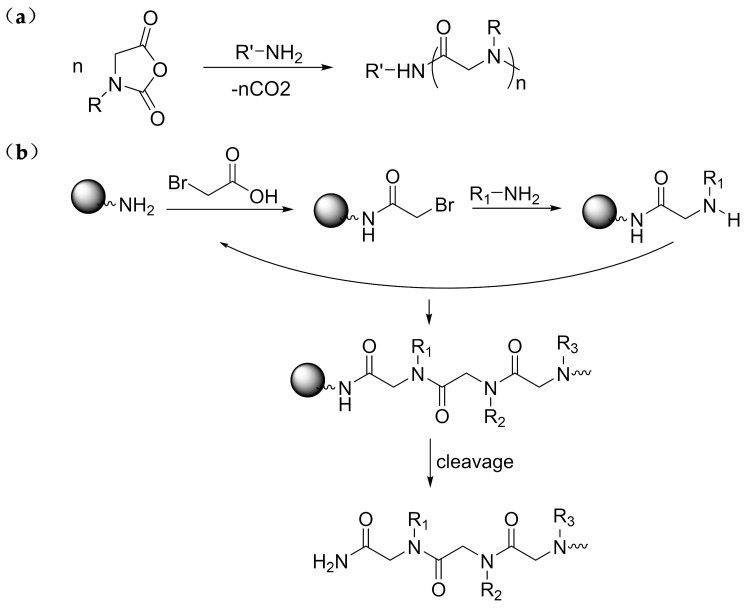
Synthesis of polypeptoid by (**a**) ring-opening polymerization of R-NCA initiated by primary amine and (**b**) solid-phase submonomer synthesis method.

There are two main synthesis methods of polypeptoids, solid-phase submonomer synthesis and ring-opening polymerization (Figure 2). For oligopeptoids, the structures can be precisely controlled by solid-phase submonomer synthesis to achieve specific responsiveness. Different reactive side chains can be introduced using various primary amines in displacement steps [61,62]. In ring-opening polymerization, primary amine and N-Heterocyclic Carbene can be used to initiate cyclic N-substituted N-carboxylic anhydride (NCA) monomers to obtain polypeptoids with longer molecular chains [63,64]. Studies have shown that polypeptoids can also be prepared from N-substituted glycine N-thiocarboxylic anhydride (NTA) monomers with primary amine as the initiator [65]. To achieve stimulus-responsive properties, multi-component reactions among isocyanates, aldehydes, acids and amines were introduced [66]. The responsive polypeptoids have different structures and compositions, including homopolymer, random copolymer, block copolymer and graft copolymer. Recently, more and more attention has been paid to the stimulus-responsive polypeptoids, which have broad applications in biomedicine and biotechnology.

### 3.1. Temperature-Responsive Polypeptoid

**Figure 3:**
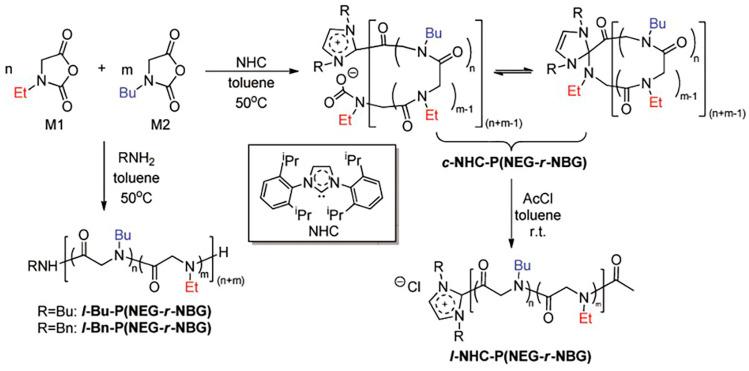
Synthesis of cyclic and linear random copoly(α-peptoid)s that have thermal-response properties in aqueous solution. Reprinted with permission from [69]. Copyright 2012, American Chemical Society.

**Figure 4:**
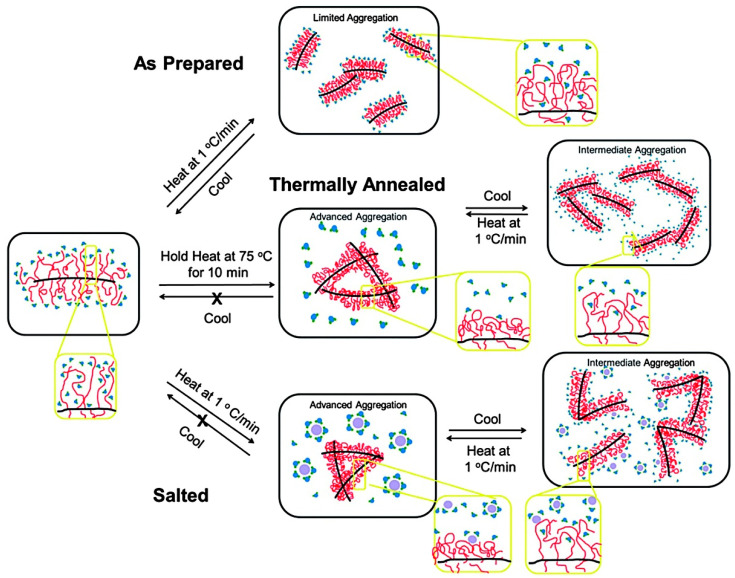
A schematic description showing the proposed polymer conformation and the level of aggregation in as-prepared or thermally annealed salt-free solutions or as-prepared salted solutions of the polypeptoid bottlebrushes. Reprinted with permission from [72]. Copyright 2014, Royal Society of Chemistry.

Polypeptoids with methyl and ethyl side chains (i.e., polysarcosine and poly (N-ethylglycine) (PNEG)) are readily soluble in water. In contrast, polypeptoids with longer alkyl side chains (with more than three carbon atoms) are proved insoluble in water. Block copolymers with hydrophilic and hydrophobic blocks can produce particular temperature response performance [67,68]. Zhang et al. used N-Heterocyclic carbene (NHC) and primary amine to initiate Et-NCA and Bu-NCA to synthesize cyclic and linear poly [(N-ethylglycine) -r-(N-butylglycine)] [P(NEG-r-NBG)] (Figure 3). The aqueous solution of the produced polypeptoid copolymers became cloudy after heating and then clear after cooling, which indicated a reversible phase transition of T_cp_. T_cp_ is defined as the temperature at 50% UV-visible transmittance (λ = 450 nm). The T_cp_ of cyclic P(NEG-r-NBG) can be adjusted in the range of 20–60 °C by changing the composition of the copolymer. However, the T_cp_ of the corresponding linear copolymer was 4−6 °C higher than that of the cyclic analog with the same composition. The reason is the entropy loss of cyclic polymer solution during phase transition is lower than that of linear polymer solution, resulting in the decrease of T_cp_ [69]. Subsequently, Zhang et al. synthesized P(NEG-r-NBG) macromonomer and then prepared corresponding polypeptoid bottle brush copolymers with different main chain length and side-chain composition by ring-opening metathesis polymerization (ROMP). The bottlebrush polymer is a kind of molecular structure with a linear polymer main chain and dense polymer side chains. The spatial repulsion brought by dense side chains can enhance the rigidity of the main chain and reduce the entanglement between molecules [70,71]. Compared with linear P(NEG-r-NBG), the T_cp_ of the synthesized bottle brush copolymer depends on the thermal history of the solution. There is no noticeable turbidity change with the increase of temperature without any treatment of the prepared bottle brush copolypeptoids. After thermal annealing of the polymer at a temperature higher than T_cp_, however, the bottle brush copolymer recovers its thermal response behavior and produce reversible T_cp_ transition. Further study indicated that the addition of inorganic salts can eliminates the dependence of T_cp_ transition on the thermal history of the solution, thus restoring the thermal response behavior (Figure 4). The results suggested that thermal annealing and salt addition can change the interactions between polymer and solvent, and are helpful for polymer conformational recombination to produce expanded to shrinked coil change. The addition of salt can enhance the synergy between local concentration of amphiphilic polypeptoid chains and hydrophobic aggregation [72]. 

**Figure 5:**
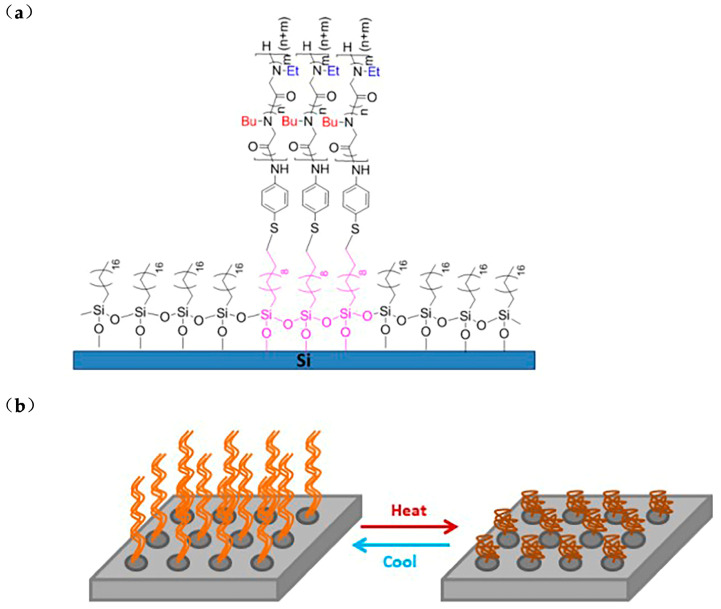
(**a**) Preparation of temperature-responsive copolypeptoids on silicon substrate; (**b**) the temperature-responsive polymer expands or collapses with the change of temperature. Reprinted with permission from [75]. Copyright 2019, American Chemical Society.

Zhang et al. synthesized linear random copolypeptoid, poly [(N-ethyl glycine) _32_-ran-(N-butyl glycine_17_)]P(NEG_32_-r-NBG_17_) and grafted it onto silicon substrate by thiol-ene click reaction (Figure 5a). Binding temperature-responsive polymer can affect the hydrophobic properties of the surface. The coating can intelligently adjust the propensity of the layer to adsorb or repel proteins or other biological macromolecules [73,74]. With the increase of temperature, the size of P(NEG_32_-r-NBG_17_) nanostructures decreases, forming a collapsed pattern. After cooling, the polymer chain stretched out to create a relaxing pattern. Surface modified with temperature-responsive polymer, whose characteristics change with temperature, can be widely used in surface-based sensor technology [75].

Molecular weight and composition of block copolymers will affect the size and shape of aggregates in solution induced by temperature change. Schlaad group synthesized a series of poly [(N-propylglycine) _x_-b-(N-methylglycine) _y_](P_x_M_y_) block copolypeptoids with different block ratios. When the temperature was higher than T_cp_, spherical micelles with a diameter of about 50 nm formed in water. After annealing and crystallization, micelles transformed into fibre intermediates and further packed into larger complex three-dimensional structures with different shapes, such as flower (P_70_M_23_), oval (P_70_M_42_ and P_70_M_76_) and irregular shapes (P_70_M_153_ and P_70_M_290_) [76]. Further study indicated that the aqueous solutions of the prepared polypeptoid copolymer have two T_cp_ but one clear point temperature (T_cl_). The two T_cp_ are between 27 °C and 45 °C, and the T_cl_ lies between the two T_cp_. The aggregation behavior of the polymer in solution was accomplished in several steps. When the first T_cp_ is reached, the hydrophobic block collapses and the polymer chains self-assemble into large structures. When the temperature is further raised to the T_cl_, the formed aggregates recombine and break into smaller micelles, resulting in clear solution. As the temperature continues to rise to the second T_cp_, micelles begin to crystallize and polymers can self-assemble into larger aggregate particles [77].

**Figure 6:**
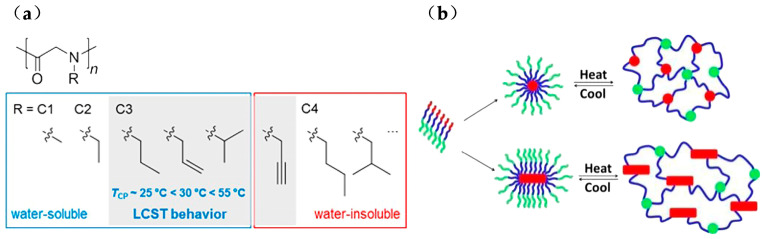
(**a**) T_cp_ range of poly (N-C3 glycine)s (C3= n-propyl, allyl, propargyl and isopropyl). Reprinted with permission from [68]. Copyright 2013, American Chemical Society. (**b**) schematic showing the proposed gelation mechanism of aqueous solutions of the triblock copolypeptoids. Reprinted with permission from [29]. Copyright 2016, American Chemical Society.

Polypeptoids can achieve temperature response by containing both hydrophilic and hydrophobic polypeptoid fragments, or having specific alkyl side chains that directly have a thermal response in aqueous solution. Schlaad group prepared a series of regular poly (N-C_3_ glycine)s (C_3_ = n-propyl, allyl, propargyl and isopropyl) with a molecular weight in the range of 1.8–6.6 kg·mol^−1^ by ring-opening polymerization. Turbidity measurement of the polymer aqueous solutions suggested that all of them showed LCST behavior except poly (N-propargylglycine). It was found that T_cp_ increases in the order of n-propyl, allyl and isopropyl as shown in Figure 6a [68]. Zhang et al. used poly (N-allylglycine) as a thermoresponsive block to synthesize triblock copolymer, which can be made into a temperature-responsive hydrogel. In low concentration (2.5–10 wt %) of water and biological medium, the copolymer can undergo sol-gel transition. The sol-gel transition was completely reversible, and the transition window was narrow. The gelation temperature (T_gel_) can be adjusted between 26.2 °C and 60.0 °C by changing the polymerization degree of different blocks. A core-shell-crown structure was formed below the T_gel_, and a hydrogel with a three-dimensional network structure was formed when the temperature rose to T_gel_ (Figure 6b). This hydrogel can be injected through the No. 24 injection needle. Related biological experiments showed that it can also induce chondrogenesis of hASCs and quantitatively encapsulate water-soluble enzymes [29]. Polypeptoids, as analogues of polypeptides, show as little cytotoxicity as peptides. Synthesis of thermoreversible hydrogel formed at human physiological temperature is very attractive for their potential applications in biological and medicine fields [78].

**Figure 7:**
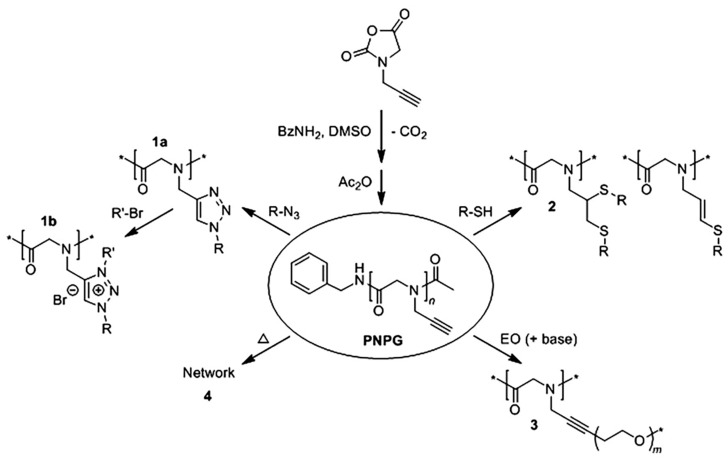
Synthesis of poly(N-propargyl glycine) and subsequent modifications of alkyne side chains. Reprinted with permission from [79]. Copyright 2015, Elsevier.

The propargyl has three carbon atoms, but the hydrophobicity of poly (N-propargyl glycine) won’t change with temperature change. The carbon-carbon triple bonds make poly (N- propargyl glycine) to be easily modified by thiol light addition, copper-catalyzed azide cycloaddition, ethylene oxide nucleophilic addition and heat-induced cross-linking. Poly (N-propargylglycine), as a modular platform, can be utilized to achieve grafted polypeptoid or ionic polypeptoid materials with specific responsiveness (Figure 7) [79]. Oligo(ethylene glycol) (OEG) moieties can show the thermal response of dehydration and hydration in water with the change of temperature [80]. The temperature-sensitive OEG unit can be introduced into the polypeptoid side chain by photoaddition and ring-opening polymerization of thioalkyne. The synthesized polymer side chain has both OEG unit and a thioether bond [81]. Compared with polyglutamate, pegylated polypeptoids have better thermal processing properties because there is no chiral center or hydrogen bond interactions in the main chain. Li et al. showed that the T_cp_ could be adjusted from 25 °C to 60 °C by changing the molecular weight and polymerization degree of OEG unit. At the same time, the thioether bond’s redox property on the side chain of the polymer can also have a more noticeable impact on the T_cp_ of the polymer, thus providing a second stimulus for the regulation of phase transition [82]. The synthesis of pegylated polypeptoids by photoaddition of mercaptan and alkyne has the disadvantages of inaccurate modification site and low efficiency. Li et al. synthesized glycosylated polypeptoid containing benzyl side chain by Schiff base and reductive amination reaction to improve this method. In the same way, the synthesized products still have thermal responsive aggregation behavior [83]. 

**Figure 8:**
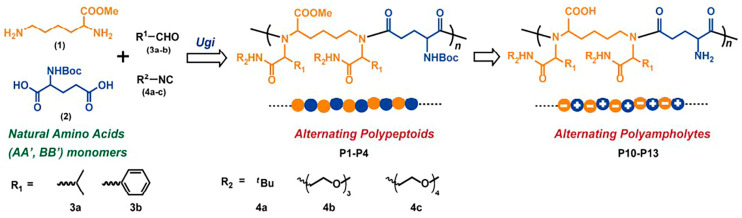
Synthesis of alternating polypeptoids and alternating polyamholytes via Ugi reaction of natural amino acids. Reprinted with permission from [84]. Copyright 2018, American Chemical Society.

Ring-opening polymerization can be used to prepare polypeptoids with long molecular chains, but it is challenging to design and synthesize polypeptoids with controllable sequences. Solid-phase submonomer synthesis method can achieve polypeptoids with absolute sequence order but suffers low efficiency. Tao et al. found that amino acids can react with aldehydes and isocyanates in sequence to form polypeptoid structures. Polypeptoids with controllable sequences can be easily synthesized by this iterative Ugi reaction of amino acids, aldehydes and isocyanates [66]. The Ugi reaction produced alternating polypeptoids with thermal response in a gradual growth mode, as shown in Figure 8. The T_cp_ of the polymer aqueous solution can be adjusted between 27 °C and 37 °C by controlling the molecular weight. Meanwhile, the molecular weight of these alternating copolymers can be as high as 15 kg·mol^−1^. The alternating copolymer synthesized by the Ugi reaction not only shows good water solubility (100 mg·mL^−1^), but also has the ability to resist protein aggregation [84].

### 3.2. PH-Responsive Polypeptoid

**Figure 9:**
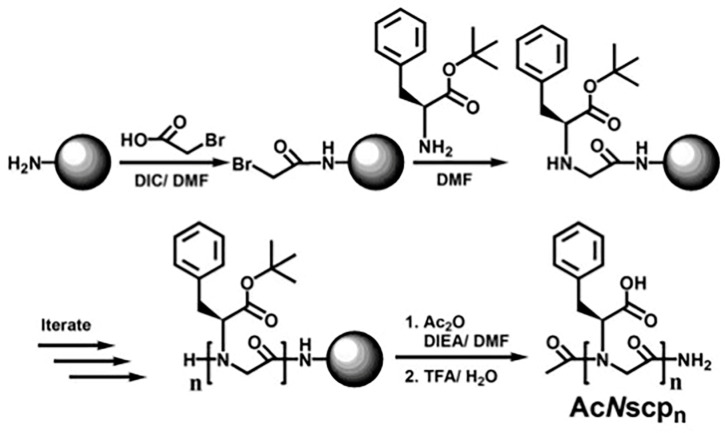
Solid-phase submonomer synthesis of AcNscpn. Reprinted with permission from [87]. Copyright 2007, American Chemical Society.

The polymers with pH responses can show the conversion of different folding states in different pH environments. The sequence-controlled pH-responsive peptoid oligomers can be prepared using a solid-phase submonomer synthesis method by repeating bromoacylation and displacement steps [85,86]. Varying functional groups, such as carboxylic acid, phosphoric acid, amine, etc., can be introduced by primary amine submonomers in displacement steps. Kirshenbaum et al. synthesized a series of (s)-N-(1-carboxyl-2-phenylethyl) glycine (Nscp) oligomers using tert-butyl L-phenylalanine as a submonomer reagent (Figure 9). The carboxylic acid in the side chain of carboxyl phenylethyl is an ionizable group, and the secondary folding structure containing the ionizing group can show pH sensitivity. This is because electrostatic interactions in different pH environment can lead to polymer conformation rearrangement [87]. 

**Figure 10:**
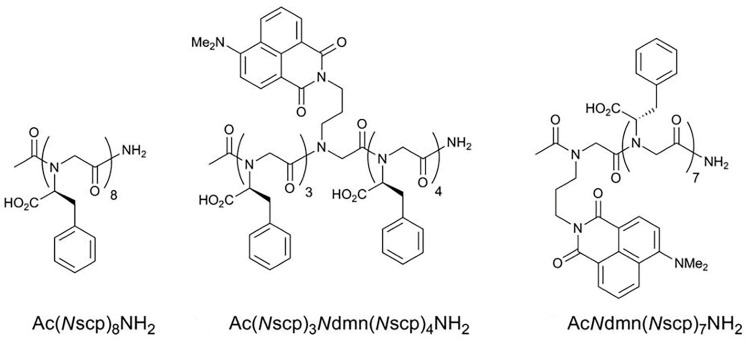
Structures of pH-responsive peptoids prepared. Reprinted with permission from [89]. Copyright 2013, John Wiley and Sons.

By combining pH-induced conformational changes with fluorescence intensity changes, polypeptoids can be used as biocompatible pH sensors. Amelia et al. labeled Nscp oligomer with the fluorescent group 4-N, N-dimethylamino-1,8-naphthalenediimide (4DMN) [88]. In different pH environments, the conformational changes caused by carboxylic acid functional groups regulate the fluorescence intensity due to the local environment change of fluorophores. When the pH value was less than 4, the protonated –COOH content was more. The polypeptoids (Figure 10) formed a compact secondary structure, which can separate the fluorescent groups from the aqueous buffer. With the increase of pH value, the fluorescence emission intensity decreased significantly. The fluorescence emission intensity can change by 24 times between pH 2.2–7, making the 4DMN labeled Nscp be applied to fluorescent polypeptoid pH sensor [89].

Polypeptoids coating can be used as surface material of hospital equipment with antifouling performance to prevent the growth of bacteria. Amelia et al. synthesized oligopeptoids containing (s)-N-(1-carboxyethyl) glycine (Nsce), (s)-n-1-(naphthylethyl) glycine (Ns1npe) and N-(2-aminoethyl glycine) (Nae). In phosphate buffer brine, the synthesized products can be adsorbed on the bare silica surface. The adsorption of water-soluble oligopeptoids on the silica surface can be affected by changing pH due to the electrostatic interactions [90,91]. Moreover, the authors introduced aromatic naphthylethyl, naphthylmethyl and phenylmethyl substituents, cationic aminoethyl substituents, neutral methoxyethyl substituents, anionic carboxymethyl and carboxyethyl substituents into the side chains of oligopeptoids. The results showed that different pH buffers could affect the polymer’s adsorption to the phospholipid membrane. By controlling these substituents’ component content and polypeptoid sequence, the interactions between polypeptoids and liposomes can change at different pH, which makes the polypeptoids applicable in the physiological pH environment [92].

Li et al. introduced amino groups into peptoid side chains through ring-opening polymerization and click chemistry. The results showed that the polymer was always soluble in water at pH ≤ 12.9. When pH increased to 13.2, LCST behavior was observed during heating. The T_cp_ of polymer decreased from 54 °C to 22 °C when pH increased from 13.2 to 13.6. Such polymers have both pH and thermal response properties and can be used in specific biomedical scenarios [35].

### 3.3. Photo-Responsive Polypeptoid

**Figure 11:**
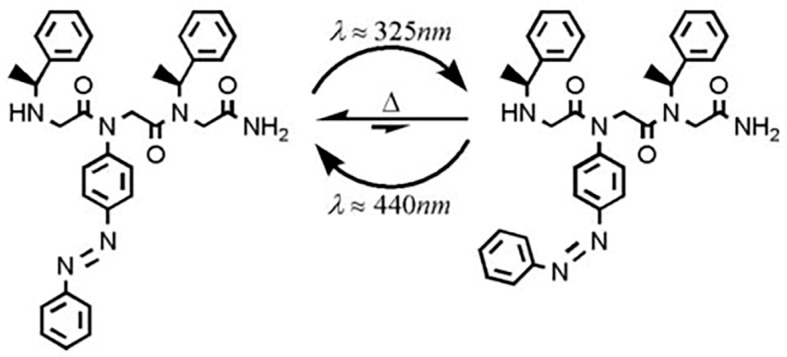
Photoisomerization and thermal back-isomerization of azobenzene peptoid. Reprinted with permission from [94]. Copyright 2008, Royal Society of Chemistry.

Light has become a valuable, and convenient external stimulus because light can be controlled in space and time and the light source is environmentally friendly and easy to obtain. Photo-responsive polypeptoid is a kind of polymer that can produce corresponding physical or chemical changes under the irradiation of light (such as ultraviolet, infrared, visible light, etc.). The main chain structure of polypeptoid is not photo-responsive. The photosensitive group can be introduced into the side chains of polypeptoid to achieve the overall photoresponse performance. These photo-responsive groups can be azobenzene, spiropyran, o-nitrobenzyl, coumarin, anthracene, cinnamic acid, thymine and diarylethene [93]. Many azobenzene functionalized polypeptoids have been shown to undergo photoinduced conformational conversion. Under the irradiation of different wavelengths of light, azobenzene groups can be converted reversibly between cis and trans conformations. Kirshenbaum Kent research group synthesized peptoid oligomers by solid-phase submonomer synthesis method and achieved photo responsiveness by doping photo-responsive azobenzene side chain. The results showed that the polymer has cis confirmation due to p–p * transition under 325 nm light and has the characteristics of trans azobenzene due to n–p * transition under 440 nm light. The photoisomerization of azobenzene does not change the skeleton conformation of peptoids, but the orientation rearrangement of the side chains occur under light (Figure 11) [94]. Zhang et al. prepared diblock polypeptoids containing azobenzene side chains by ring-opening polymerization. The diblock copolymer assembled into different morphologies by adjusting the polymer components, including spherical and rod-like micelles. Furthermore, it was found that the morphological transformation of the polymer under alternating ultraviolet–visible (UV-vis) illumination is reversible [95]. The photoinduced anisotropy of azobenzoylated polypeptoids has essential application prospects in optical information storage, optically controlled molecular orientation, molecular switch and integrated optics.

**Figure 12:**
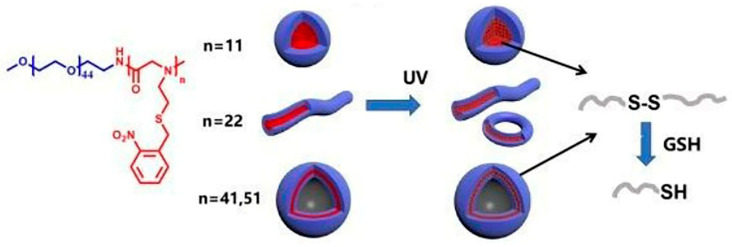
The reversible crosslinking assemblies from photo-responsive polypeptoids. Reprinted with permission from [98]. Copyright 2020, Royal Society of Chemistry.

O-nitrobenzyl group is stable in an acidic and alkaline environment and has highly controllable photochemical properties [96,97]. Li et al. synthesized a diblock copolymer PEG-b-poly (n-(s- (o-nitrobenzyl)-thioethyl) glycine) (PEG-b-PNSN) by ring-opening polymerization. Under the irradiation of ultraviolet light, o-nitrobenzyl group was photolyzed to produce a free mercaptan group, which spontaneously oxidized to form disulfide bond. By controlling the chain length of PNSN, the diblock copolymers self-assembled into different aggregates. With the increase of polymerization degree of hydrophobic block PNSN, the polymer morphology changed from spherical, short columnar to vesicular. The cross-linking of the disulfide bond formed by photooxidation can keep the nanostructure stable. Simultaneously, in the presence of reducing agent glutathione (GSH), the spontaneous oxidation of the thiol groups to disulfide bonds under ultraviolet irradiation was reversible, which made the cross-linking process reversible, as shown in Figure 12 [98]. The nitrobenzyl-modified polypeptoids have great potential applications as photo-controlled drug delivery materials, functional gels and photoelectric sensors [99].

### 3.4. Redox- Responsive Polypeptoid

**Figure 13:**
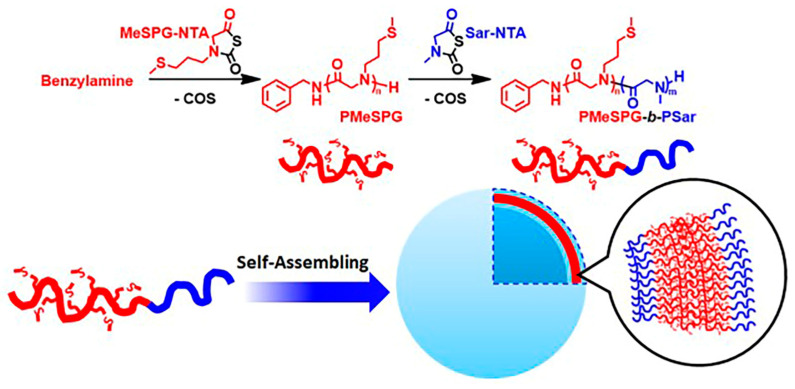
Synthesis of PMeSPG-b-PSar block copolymers and their self-assembly. Reprinted with permission from [103]. Copyright 2019, American Chemical Society.

Compared with normal cells, there are higher concentrations of reactive oxygen species in tumor and inflamed tissues [100,101,102]. This particular redox microenvironment can be used in the design of responsive drug delivery systems. Usually, polymers sensitive to oxidation include polymers containing selenium, tellurite, aryl oxalate, polysulfide and phenylborate. Ling et al. synthesized redox responsive diblock copolymers containing hydrophilic segment polysarcosine (Psar) and hydrophobic segment poly (N-3- (methylthio) alanine) (PMeSPG). The hydrophobic thioether side chain of PMeSPG can be transformed into a hydrophilic sulfoxide side chain in the presence of reactive oxygen, as shown in Figure 13 [103]. Redox-responsive polypeptoid has broad application prospects in the related fields of cancer chemotherapy drug delivery.

## 4. Conclusions

This review discussed the structural design and functional applications of different responsive materials constructed from polypeptoid frameworks. Polypeptoids have excellent biological properties such as low cytotoxicity and good biocompatibility, making researchers constantly introduce mature, responsive units to achieve their responsive properties. Many researchers have designed and prepared different polypeptoid structures responsive to environmental changes (such as temperature, pH, UV/Vis, ionic strength and reactive oxygen species). However, the research mainly focuses on temperature response and pH response, and other responsive polypeptoids are still underexplored. According to the different responsiveness, polypeptoids can be used in drug delivery, tissue engineering, antifouling coatings for medical devices, intelligent surfaces, biosensors, etc. In the past ten years, responsive polypeptoid materials have been continuously developed. Polypeptoids show a wide application prospect in many fields, but it is still difficult to be commercialized or go to the clinical trial stage. Future work includes introducing more responsive units into polypeptoid side chains to find more suitable molecular structures for different specific scenarios. Stimuli-reponsive polypeptoids can also be combined with DNA, glycoprotein and other materials to accelerate their clinical applications.
